# Supplementary material for: Single Cell Transcriptomic and Chromatin Profiles Suggest Layer Vb Is the Only Layer With Shared Excitatory Cell Types in the Medial and Lateral Entorhinal Cortex
Source: Front Neural Circuits. 2022 Jan 26;15:806154. doi: 10.3389/fncir.2021.806154 (PMC8826650; doi:10.3389/fncir.2021.806154)
Supplement: Supplementary file 14 [file Data_Sheet_1.ZIP › LEC_Sup_vs_MEC_Sup_Motifs/homerResults/motif19.similar.html]

motif19

## Information for motif19

G
T
A
C
G
C
T
A
C
G
A
T
G
T
A
C
G
T
C
A
C
G
T
A
G
T
A
C
G
C
T
A
T
G
A
C
G
T
A
C
G
T
C
A
C
G
T
A
  
Reverse Opposite:  

G
C
A
T
C
A
G
T
C
A
T
G
A
C
T
G
C
G
A
T
A
C
T
G
G
C
A
T
C
A
G
T
C
A
T
G
G
C
T
A
C
G
A
T
C
A
T
G
  

|  |  |
| --- | --- |
| p-value: | 1e-14 |
| log p-value: | -3.371e+01 |
| Information Content per bp: | 1.592 |
| Number of Target Sequences with motif | 2493.0 |
| Percentage of Target Sequences with motif | 18.70% |
| Number of Background Sequences with motif | 3217.5 |
| Percentage of Background Sequences with motif | 16.15% |
| Average Position of motif in Targets | 328.2 +/- 235.6bp |
| Average Position of motif in Background | 357.0 +/- 271.6bp |
| Strand Bias (log2 ratio + to - strand density) | -0.0 |
| Multiplicity (# of sites on avg that occur together) | 1.15 |
| Motif File: | file (matrix) reverse opposite |

### Similar de novo motifs found

|  |  |  |  |  |  |  |  |
| --- | --- | --- | --- | --- | --- | --- | --- |
| Rank | Match Score | Redundant Motif | P-value | log P-value | % of Targets | % of Background | Motif file |
| 1 | 0.731 | A C G T A C T G A C G T A C G T A C T G C G T A A C G T A C T G C G T A A C G T | 1e-12 | -29.243881 | 0.28% | 0.07% | motif file (matrix) |
| 2 | 0.709 | G A C T A T C G A G C T A C G T A C T G A C G T A G C T A C T G A C G T A C G T | 1e-7 | -16.662086 | 5.46% | 4.48% | motif file (matrix) |
